# Hepatitis B virus induced cytoplasmic antineutrophil cytoplasmic antibody-mediated vasculitis causing subarachnoid hemorrhage, acute transverse myelitis, and nephropathy: a case report

**DOI:** 10.1186/s13256-017-1255-x

**Published:** 2017-04-03

**Authors:** Utsav Joshi, Roshan Subedi, Bikram Prasad Gajurel

**Affiliations:** Tribhuvan University, Institute of Medicine, Maharajgunj Medical Campus, Kathmandu, Nepal

**Keywords:** Hepatitis B, Vasculitis, Subarachnoid hemorrhage, Transverse myelitis, Nephropathy

## Abstract

**Background:**

Transverse myelitis, subarachnoid hemorrhage, and nephropathy are established but rare complications of hepatitis B virus infection that can potentially be triggered by an antibody-mediated vasculitis as a result of a viral infection. The following is a case report detailing a patient presenting with all three of the above presentations who is cytoplasmic antineutrophil cytoplasmic antibody-positive and a chronic carrier of hepatitis B.

**Case presentation:**

A 33-year-old Nepalese man presented to our hospital with headache, swelling of his body, paraplegia, and back pain that developed over a period of 10 days. Laboratory studies showed proteinuria and elevated levels of serum urea and creatinine. Viral serology was suggestive of chronic inactive hepatitis B carrier state. A computed tomography scan of his head revealed features suggestive of subarachnoid hemorrhage. Magnetic resonance imaging of his dorsal spine showed diffuse T2 high signal intensity within his spinal cord extending from second to 12th thoracic vertebral level which was suggestive of transverse myelitis. The origin of these symptoms was attributed to immune complex-mediated vasculitis after serum analysis for cytoplasmic antineutrophil cytoplasmic antibody came out positive. He was managed with steroids administered orally and intravenously and entecavir administered orally.

**Conclusion:**

This case highlights the possibility of a hepatitis B virus-induced vasculitis as the cause of subarachnoid hemorrhage, transverse myelitis, and nephropathy.

## Background

Hepatitis B is an established cause of chronic liver disease and hepatocellular carcinoma [1]. It is associated with several extrahepatic manifestations including but not limited to serum sickness, polyarteritis nodosa, urticaria, leukocytoclastic vasculitis, and essential mixed cryoglobulinemia. In addition to these manifestations, hepatitis B virus (HBV) has also been documented to be involved in the etiopathogenesis of antineutrophil cytoplasmic antibodies (ANCA)-positive vasculitis [2].

HBV-induced vasculitis is an uncommon but important cause of subarachnoid hemorrhage (SAH), transverse myelitis, and nephropathy. Common causes of SAH include aneurysm, arteriovenous malformations, anticoagulation, and brain tumors, possibly followed by vasculitis [3]. Transverse myelitis, on the other hand, is an inflammation of the spinal cord manifesting as weakness, sensory loss, and autonomic dysfunction. Although a large proportion of cases of transverse myelitis are idiopathic in origin, quite a few cases have been attributed to vasculitis in addition to conditions like infectious, parainfectious, and systemic autoimmune diseases, and paraneoplastic and ischemic disease [4]. HBV has also been widely recognized to be associated with a wide variety of glomerulonephritis, notably membranous nephropathy [5]. Other causes include immunological disorders, inherited diseases, metabolic diseases, and deposition disorder [6].

We report an interesting case of SAH, transverse myelitis, and nephrotic syndrome in a patient who is a chronic carrier of hepatitis B.

## Case presentation

A 33-year-old Nepalese man presented to our department of neurology in a university hospital for evaluation of an acute-onset flaccid paraplegia in the background of a 10-day history of headache and puffiness of the face.

Ten days prior to his presentation to our hospital, he had sudden onset, unilateral right-sided headache that later became generalized. He developed facial puffiness concomitant with headache that gradually progressed to involve his lower limbs. This was associated with shortness of breath on exertion, but without cough, fever, and decreased urinary output. For these complaints, he was initially evaluated at a local medical center. During his stay at the center, on his eighth day of admission, he developed stiffness of the neck for which he underwent lumbar puncture. The lumbar puncture yielded a bloody tap. He was then referred to our center for further evaluation.

During his transfer from the local medical center to our hospital, he developed a sudden onset weakness of both lower limbs with loss of sensation. He presented with all of these symptoms at our center 10 days after the onset of headache. The loss of sensation gradually spread up to the level of his xiphisternum. He also developed severe back pain in his mid-thoracic region. He gave no history of weakness or any sensory disturbances involving his upper limbs. However, there was a history of both bowel and urinary incontinence. There was no history of loss of consciousness, altered sensorium, or seizures. He did not have any ocular symptoms at presentation. There was no evidence of a recent respiratory or gastrointestinal tract infection prior to the development of lower limb weakness. He had no history of alcohol intake or tobacco smoking. There was also no history of chronic medical illness.

His higher mental functions and cranial nerves examination were normal. Visual acuity and fundal examination were also normal. Muscle strength was intact in his upper limbs. Muscle tone was decreased in both lower limbs. Power across all major muscle groups in his lower limbs was 0/5. Deep tendon reflexes were absent in his lower extremities. Bilateral plantar responses were mute. A sensory examination revealed a sensory level at approximately T4/T5.

Blood investigations showed: hemoglobin 12.1 gm/dl, packed cell volume 36%, white blood cells 17190/mm^3^ (neutrophil 82%, lymphocytes 16%, eosinophils 1%), and platelets 213,000/mm^3^. His renal function test was deranged: urea 21 mmol/l and creatinine 185 μmol/l. Urine routine evaluation showed: white blood cells 1 to 2 cells per high power field, red blood cells 8 to 10 cells per high power field, and albumin 3+ without any casts. Tests for human immunodeficiency virus (HIV) and hepatitis C virus (HCV) were negative. Serology was positive for hepatitis B surface antigen (HBsAg) and hepatitis B e antibodies (anti-HBe) while hepatitis B e antigen (HBeAg) and hepatitis B core IgM antibodies (anti-HBc IgM) were negative. His hepatitis B viral load was less than 2000 IU/ml. Venereal Disease Research Laboratory (VDRL) test and rapid plasma reagin test were negative. Serology was negative for cytomegalovirus (CMV), varicella zoster virus (VZV), and herpes simplex virus (HSV). His 24-hour urinary total protein was 3.9 grams per day, suggestive of nephrotic range proteinuria.

Cerebrospinal fluid (CSF) analysis revealed pleocytosis with elevated protein and normal glucose: total count 1300 with 70% polymorphs and 30% monomorphs, protein 380 mg/dl, and sugar 5.3 mmol/l. The red blood cells count in CSF was 12,500/mm^3^ and the opening pressure was 26 cm of water. Microscopy with gram stain and acid-fast bacilli stain followed by culture did not reveal any organism. CSF VDRL was also negative.

**Fig. 1:**
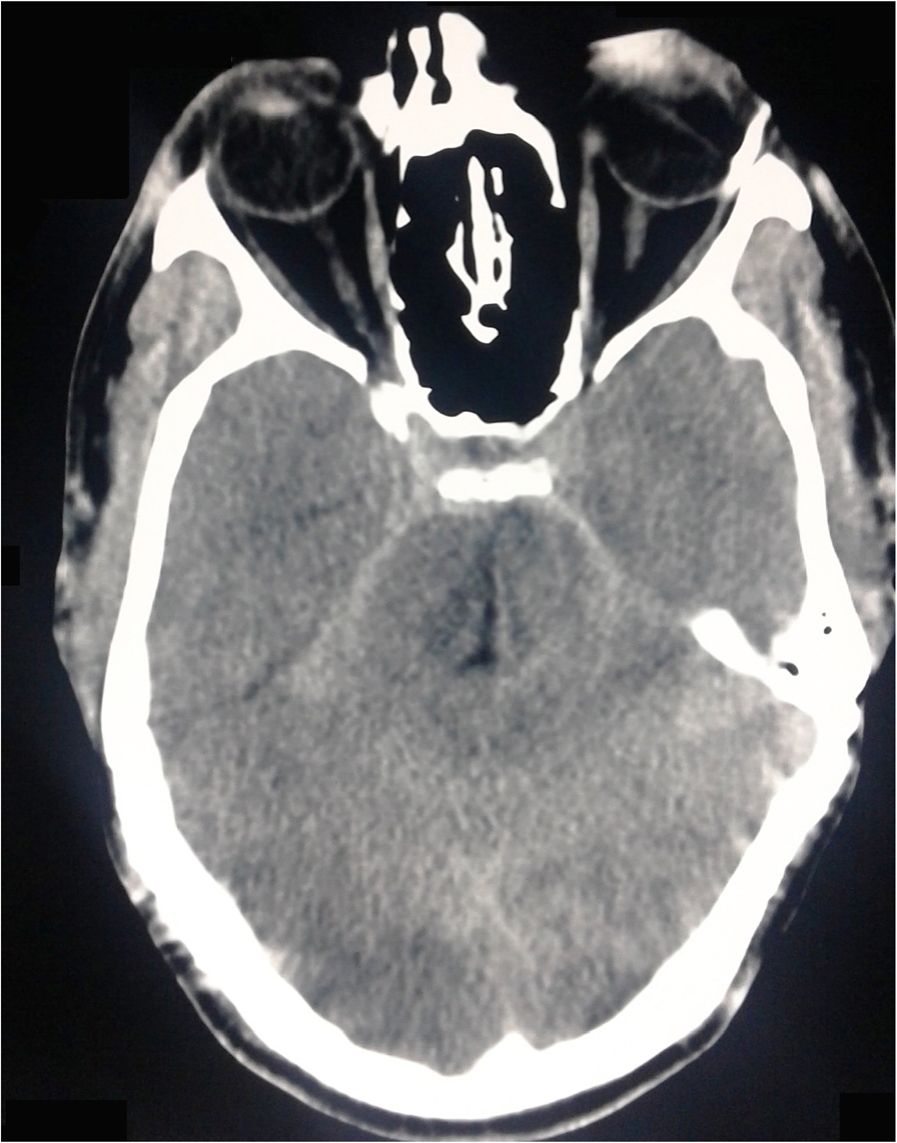
Computed tomography image of the head; the axial section of the brain is shown. The image shows linear hyperdense areas in the ambient and suprasellar cisterns suggestive of subarachnoid bleeding

**Fig. 2:**
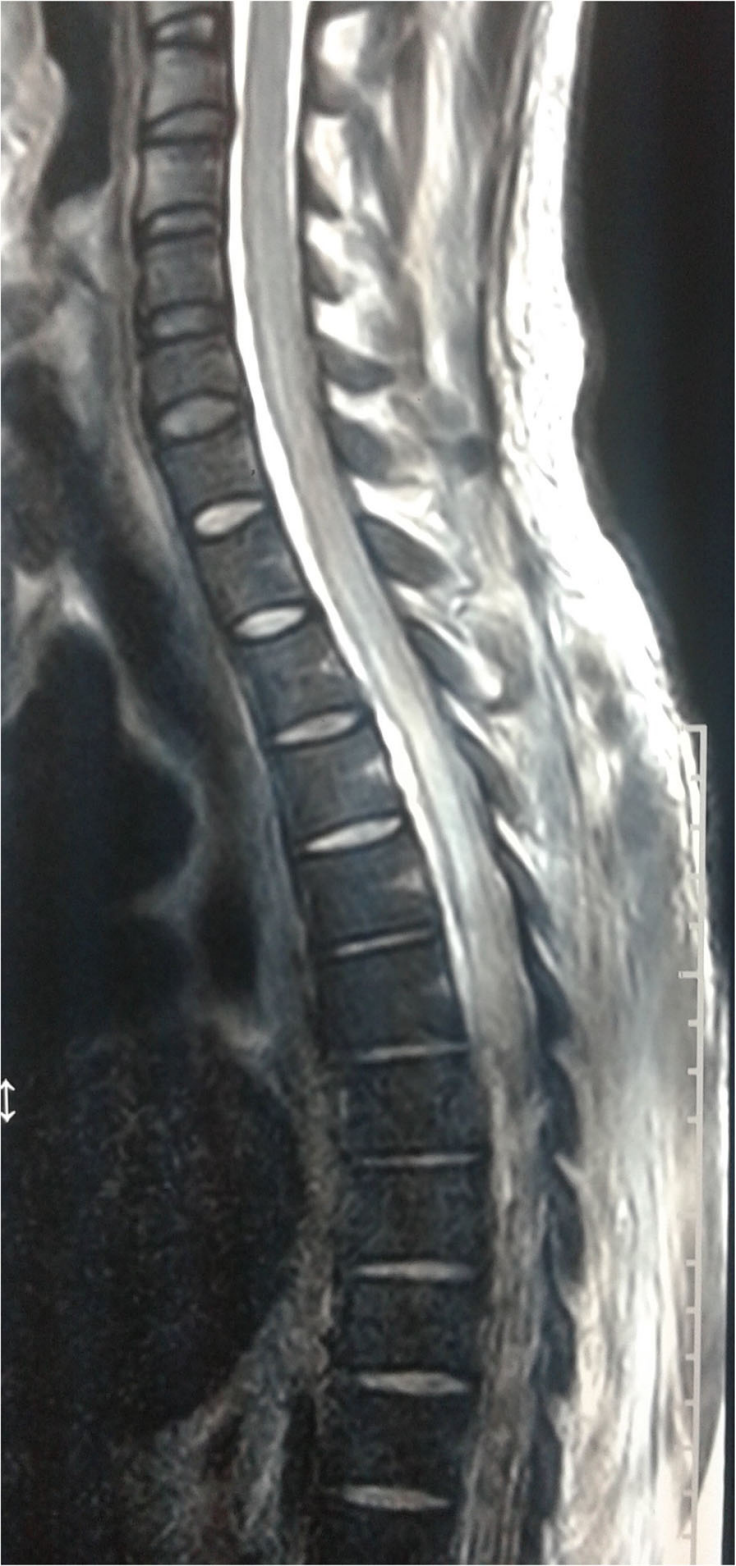
Magnetic resonance image of thoracic spine; sagittal view of thoracic segment of the spinal cord is shown. The image shows diffuse T2 high signal intensity within the spinal cord extending from second to 12th thoracic vertebral level

A computed tomography (CT) scan of his head revealed linear hyperdense areas in his ambient and suprasellar cisterns which was suggestive of SAH (Fig. 1). Magnetic resonance imaging (MRI) of his dorsal spine showed diffuse T2 high signal intensity within his spinal cord extending from second to 12th thoracic vertebral level which was suggestive of transverse myelitis (Fig. 2). A CT cerebral and spinal angiogram did not reveal any abnormalities. However, an MRI of his brain was not done.

Serological evaluations for antinuclear antibodies and anti-double stranded-deoxyribonucleic acid antibodies were negative and his serum angiotensin-converting enzyme (ACE) level was normal. Immunofluorescent assay showed perinuclear-ANCA (p-ANCA) within range but cytoplasmic-ANCA (c-ANCA) was positive with end-point titer over 1:20. In the light of positive serological tests for HBV and c-ANCA that pointed toward the vasculitic origin of myelitis, a further test for neuromyelitis optica (NMO) was not carried out.

The final impression was that of a SAH with acute transverse myelitis and with nephrotic syndrome in a patient who is a chronic carrier of hepatitis B. He was started on entecavir administered orally for hepatitis B infection. He was treated for myelitis with methylprednisolone administered intravenously (1 gram per day) for 3 days followed by a short course of prednisolone administered orally. During the course of his stay at our hospital, his sensory symptoms improved but his motor symptoms did not improve. Moreover, it was not economically feasible for him to remain in our hospital any longer and, hence, he was discharged after 1 month of hospital stay. He and his family members were counselled about rehabilitative care to prevent secondary complications of prolonged immobility. He came for regular follow-up in our neurology clinic for the next 3 months during which his condition remained the same with no improvement in motor function. After 3 months, he was lost to follow-up.

## Discussion

This case report describes an atypical case of SAH, transverse myelitis and nephrotic syndrome in an HBsAg-carrier.

Infectious agents can be the underlying cause of several vasculitides. Various viruses have been found to be responsible for vasculitis: HCV, HBV, HIV, CMV, VZV, HSV, and human T-cell lymphotropic virus type 1 (HTLV-1) to name a few of them [7]. Extrahepatic manifestations due to HBV infection may be attributed to deposition of immune complexes. Immune complexes promote aggregation of platelets and activation of Hageman factor that result in inflammation and microthrombi formation. Deposition of these complexes in small arteries and glomeruli may be responsible for clinical presentations of vasculitis and nephritis [8]. In the literature, a causal relationship has only been firmly established in a few instances of vasculitis, such as chronic HBV infection and polyarteritis nodosa [9].

Immune complex-mediated vasculitis is also associated with ANCA positivity. A positive association has been demonstrated between spinal cord lesion and ANCA; especially between longitudinally extensive transverse myelitis and ANCA. A significant number of patients with transverse myelitis have been found to be p-ANCA and c-ANCA positive, albeit these cases were idiopathic [10]. Moreover, borderline positive c-ANCA was significantly higher in chronic hepatitis B group as compared to control [2].

Vasculitis associated with HBV infection has been reported, although rarely, to be responsible for central and peripheral nervous system lesions including myelitis [11]. It may play an important role in the pathogenesis of transverse myelitis, indirectly by triggering an immune reaction that damages neural tissue. This immunological phenomenon is further supported by the observation that the preceding infection fully subsides before the onset of symptoms and no infectious agent can be detected in the central nervous system. Cases of transverse myelitis subsequent to vaccination have also been reported [4]. The same immunological phenomenon secondary to HBV infection might be the cause of SAH in our patient. Even though aneurysms are the most common cause of non-traumatic SAH, vasculitis together with arteriovenous malformations, anticoagulation, and brain tumor should also be considered to be other important causes of SAH [3].

Besides neurological involvement, membranous nephropathy is one of the widely recognized forms of glomerulonephritis associated with HBV infection [5]. Subendothelial and mesangial immune deposits may cause capillary and mesangial injuries with subsequent inflammation and nephropathy [12]. The same pathogenesis may be used to explain the nephrotic-range proteinuria and renal impairment in our patient.

In our case, the patient did not have any clinical features suggestive of optic neuritis. Although myelitis alone is sufficient to make a diagnosis of NMO, the seropositivity for HBV and c-ANCA pointed toward the vasculitic origin of transverse myelitis and gave a plausible explanation for the occurrence of myelitis. Moreover, the economic situation of our patient and the cost of the test had to be taken into account in this case and, for the same reason, it would not have been cost effective or helpful. Besides, MRI of the brain was also not done in our patient. A CT scan of his head showed typical SAH and the MRI findings of his dorsal spine combined with SAH could be explained by HBsAg-induced vasculitis. In the light of these results, an MRI of his brain was also deemed unnecessary.

## Conclusions

This case report highlights the possibility of HBV infection as a cause of vasculitis. Immune complex-mediated vasculitis triggered by chronic hepatitis B infection might result in myelitis along with SAH and nephropathy. Physicians should not overlook the possibility of neurological and renal symptoms being triggered by HBV infection in a patient who is a chronic carrier.

## Abbreviations

ACE: Angiotensin-converting enzyme
ANCA: Antineutrophil cytoplasmic antibody
Anti-HBc IgM: Hepatitis B core IgM antibodies
Anti-HBe: Hepatitis B e antibodies
c-ANCA: Cytoplasmic antineutrophil cytoplasmic antibody
CMV: Cytomegalovirus
CSF: Cerebrospinal fluid
CT: Computed tomography
HBeAg: Hepatitis B e antigen
HBsAg: Hepatitis B surface antigen
HBV: Hepatitis B virus
HCV: Hepatitis C virus
HIV: Human immunodeficiency virus
HSV: Herpes simplex virus
HTLV-1: Human T-cell lymphotropic virus type 1
MRI: Magnetic resonance imaging
NMO: Neuromyelitis optica
p-ANCA: Perinuclear-antineutrophil cytoplasmic antibody
SAH: Subarachnoid hemorrhage
VDRL: Venereal Disease Research Laboratory
VZV: Varicella zoster virus

## References

[1] Anstee QM, Jones DEJ. Liver and biliary tract disease. In: Walker BR, Colledge NR, Ralston SH, Penman I, editors. Davidson’s Principles and Practice of Medicine. 22nd ed. Toronto: Elsevier Health Sciences; 2013. p. 921–88.

[2] Calhan T, Sahin A, Kahraman R, Altunoz ME, Ozbakir F, Ozdil K, Sokmen HM. Antineutrophil Cytoplasmic Antibody Frequency in Chronic Hepatitis B Patients. Dis Markers. 2014;2014:982150. 10.1155/2014/982150 PMC4138952 25161336

[3] Sveinsson ÓÁ, Ólafsson IH, Kjartansson Ó, Valdimarsson EM. Spontaneous subarachnoid haemorrhage – review. Laeknabladid. 2011;97(6):355–62. 10.17992/lbl.2011.06.377 21659676

[4] Borchers AT, Gershwin ME. Transverse myelitis. Autoimmun Rev. 2012;11(3):231–48. 10.1016/j.autrev.2011.05.018 21621005

[5] Zhou TB, Jiang ZP. Is there an association of hepatitis B virus infection with minimal change disease of nephrotic syndrome? A clinical observational report. Ren Fail. 2015;37(3):459–61. 10.3109/0886022X.2014.1001711 25585950

[6] Goddard J, Turner AN. Kidney and urinary tract disease. In: Walker BR, Colledge NR, Ralston SH, Penman I, editors. Davidson’s Principles and Practice of Medicine. 22nd ed. Toronto: Elsevier Health Sciences; 2013. p. 461–524.

[7] Pagnoux C, Cohen P, Guillevin L. Vasculitides secondary to infections. Clin Exp Rheumatol. 2006;24(2):S71–81. 16859600

[8] Singh H, Tanwar VS, Sukhija G, Kaur P, Govil N. Vasculitis as a Presenting Manifestation of Chronic Hepatitis B Virus Infection: A Case Report. J Clin Diagn Res. 2016;10(2):OD25–6. 10.7860/JCDR/2016/17384.7304 PMC4800578 27042512

[9] Teng GG, Chatham WW. Vasculitis related to viral and other microbial agents. Best Pract Res Clin Rheumatol. 2015;29(2):226–43. 10.1016/j.berh.2015.05.007 26362741

[10] Long Y, Zheng Y, Chen M, Zhang B, Gao C, Gao Q, Yin JR, Pu S, Xie C. Antineutrophil Cytoplasmic Antibodies in Patients with Idiopathic Inflammatory-Demyelinating Diseases. Neuroimmunomodulation. 2014;21(6):297–303. 10.1159/000357681 24642629

[11] Guillevin L, Mahr A, Callard P, Godmer P, Pagnoux C, Leray E, Cohen P. French Vasculitis Study Group. Hepatitis B virus-associated polyarteritis nodosa: clinical characteristics, outcome, and impact of treatment in 115 patients. Medicine. 2005;84(5):313–22. 10.1097/01.md.0000180792.80212.5e 16148731

[12] Fukuoka K, Nakabayashi K. Secondary nephrotic syndrome due to collagen disease and vasculitis. Nihon Rinsho. 2004;62(10):1898–906. 15500137

